# 不同胸外科医生主刀进行肺癌手术的患者生存差异分析

**DOI:** 10.3779/j.issn.1009-3419.2018.02.08

**Published:** 2018-02-20

**Authors:** 宁 李, 锋维 谭, 斌 邱, 嘉根 李, 峻 赵, 禹舜 高, 大力 王, 友生 毛, 奇 薛, 巨伟 牟, 树庚 高, 捷 赫

**Affiliations:** 100021 北京, 国家癌症中心/中国医学科学院北京协和医学院肿瘤医院胸外科 Department of Thoracic Surgery, National Cancer Center/Cancer Hospital, Chinese Academy of Medical Science and Peking Union Medical College, Beijing 100021, China

**Keywords:** 肺肿瘤, 外科, 手术, 医生, 预后, Lung neoplasms, Surgery, Operation, Surgeon, Prognosis

## Abstract

**背景与目的:**

外科医生是肺癌患者手术治疗的直接决策者和执行者，是否医生间的差异会影响到患者生存尚不清楚。本研究分析不同的胸外科医生手术治疗的患者5年生存率，评估医生的影响和作用。

**方法:**

回顾性分析2002年-2007年5年间，在中国医学科学院肿瘤医院胸外科进行手术治疗的肺癌患者。依据不同主刀医生进行分组，比较患者基本信息、手术方式、短期结果和长期生存之间的差异。

**结果:**

共有11位经验丰富的胸外科医生主刀治疗的712例患者纳入本研究。各位医生诊治患者的性别、年龄、吸烟、病理类型间无明显差异。而在临床分期、手术方式、手术时间、术中输血率、淋巴结清扫个数和姑息性切除比例、术后严重并发症发生率、围手术期死亡率等方面都存在显著差异。不同医生治疗患者5年生存率存在显著差异，这种差异在各临床期别分析中均可见到，具有一致性。多因素分析中提示主刀医生是影响患者预后生存的独立因素。

**结论:**

主刀医生对于肺癌患者的治疗效果存在显著影响。

## Abstract (en)

**Background and objective:**

Surgeons are the direct decision-makers and performers in the surgical treatment of patients with lung cancer. W hether the differences among doctors affect the sur vival of patients is unclear. This study analyzed the five-year sur vival rates of different thoracic surgeries in patients undergoing surgery to assess the physician's impact and impact.

**Methods:**

A retrospective analysis of five years between 2002-2007 in the Department of Thoracic Surgery, Cancer Hospital, Chinese Academy of Medical Sciences, for surgical treatment of lung cancer patients. According to different surgeons grouping doctors to compare the basic information of patients, surgical methods, short-term results and long-term survival differences.

**Results:**

A total of 712 patients treated by 11 experienced thoracic surgeons were included in this study. The patients have nosignificant difference with gender, age, smoking, pathological type between groups. There were significant differences in clinical staging, surgery type, operation time, blood transfusion rate, number of lymph node dissection, palliative resection rate, postoperative complications and perioperative mortality. There was a significant difference in five-year survival rates among patients treated by different doctors. This difference can be seen in all clinical stage analyzes with consistency. In the multivariate analysis, it was suggested that surgeon was an independent factor influencing the prognosis of patients.

**Conclusion:**

Thoracic surgeon has a significant effect on the therapeutic effect of lung cancer patients.

手术治疗是肺癌治疗的关键步骤^[1]^，而主刀医生是这一步骤的直接决策者和执行者^[2]^。由于外科医生个人对于手术理念、患者选择、技术把握、操作习惯等多方面都存在区别；行业间也承认不同医生手术视觉水平存在一定差异^[3, 4]^。但是在依从国际通行指南的基础上，外科医生个体间的差异，是否会影响到患者治疗效果，始终是一个存在争议的地带，尚无相关报道。本研究采用回顾性分析2002年-2007年间我院手术治疗的非小细胞肺癌患者，依据不同主刀医生进行分组，分析外科医生对于肺癌患者治疗结果的影响。

## 1 资料与方法

### 1.1 病例资料

2003年1月1日-2007年12月31日5年间，在中国医学科学院肿瘤医院胸外科接受手术的非小细胞肺癌患者。

### 1.2 纳入标准

① 接受我院有经验主刀医师治疗的患者；②经病理确认为非小细胞肺癌；③临床资料完整；④随访资料完整。

### 1.3 主刀医师纳入标准

获得副主任医师及以上职称，肺癌手术经验在100台次以上，本次研究纳入病例数超过30例。

### 1.4 研究方法

本研究为回顾性研究，收集患者的性别、年龄、吸烟史、肿瘤类型、病理分期、手术方式、手术时间、根治性切除率、淋巴结清扫个数、术中输血率、围手术期死亡率、术后生存时间等信息。本研究经过中国医学科学院肿瘤医院伦理委员会批准。

### 1.5 统计学分析

采用SPSS 13.0软件进行处理，针对组间计数和计量资料差异采用*t*检验、卡方检验分析；针对患者生存时间，采用*Cox*多因素回归、*KM*生存曲线方法进行统计分析。*P* < 0.05为差异有统计学意义。

## 2 结果

### 2.1 患者基本资料

共有712例非小细胞肺癌患者符合条件纳入本项研究，并依据不同主刀医师分为11个组别中：S1-S11。患者各组间性别、年龄、吸烟史、病理类型未见明显差异。但病理分期存在显著差异（*P* < 0.01）（表 1）。

**1:** 患者基本资料 Characteristics of patients

Group	S1	S2	S3	S4	S5	S6	S7	S8	S9	S10	S11	Total
Number	136	117	111	65	59	49	41	36	35	32	31	712
Gender												*P*=0.69
Male	104	88	92	48	44	39	28	27	29	26	21	
Female	32	29	19	17	15	10	13	9	6	6	10	
Age (yr)												*P*=0.29
Average	59.5	59.5	59.7	61.3	61.6	59.6	63	59.1	61.1	58.2	61.7	
Median	60	60	60	60	64	61	64	58	62	59	63	
Range	30-81	33-81	33-82	38-81	33-75	33-74	46-79	40-76	42-75	39-75	41-77	
Smoking history												*P*=0.34
Smoke	86	71	84	43	43	37	25	23	24	24	20	
No smoke	50	46	27	22	16	12	16	13	11	8	11	
Pathology												*P*=0.13
SCC	63	49	51	37	31	29	26	24	17	18	15	
AD	73	68	60	28	28	20	15	12	18	14	16	
Pathology stage												*P* < 0.01
Stage Ⅰ	51	60	43	34	21	15	21	19	21	13	12	
Stage Ⅱ	22	29	12	13	19	10	6	10	7	7	6	
Stage Ⅲ	63	28	56	18	19	24	14	7	7	12	13	
SCC: squamous cell lung cancer; AD: adenocarcinoma.												

### 2.2 患者手术资料

经统计手术方式可见，肺叶切除术为各主刀医生最常见的手术方式，占全部手术的67.4%（49%-77%）。全肺切除术和袖状切除术在各术者中差异最大。总体术式选择存在一定差异（*P*=0.06）；手术时间从最快术者平均2.1 h到最慢竖着3.5 h存在显著差异；术中输血比例最高24.5%，最低2.6%，存在显著差异；淋巴结清扫数量最多组28.4个，最低组16.7个，存在显著差异（表 2）。

**2:** 患者手术资料 Operation details of patients

Group	S1	S2	S3	S4	S5	S6	S7	S8	S9	S10	S11	Total
Number	136	117	111	65	59	49	41	36	35	32	31	712
Method												*P*=0.06
Thoracotomy exploration	0	1	2	2	0	0	0	0	0	0	0	
Wedge resection	0	1	1	0	0	1	0	0	0	0	0	
Lobectomy	87	88	78	42	37	24	31	24	25	20	24	
Compound lobectomy	9	6	10	4	8	8	3	4	4	2	5	
Pneumonectomy	28	17	17	12	8	13	6	7	5	10	1	
Sleeve resection	11	2	2	5	6	2	1	1	1	0	1	
Carina angioplasty	1	2	1	0	0	1	0	0	0	0	0	
Operation (h)												
Average	3.1	2.5	3	2.4	3	3.5	3.1	3.1	3	3	2.1	
Range	73	68	60	28	28	20	15	12	18	14	16	
Intraoperative blood transfusion												*P* < 0.001
Yes	22	3	15	2	2	12	2	7	1	5	1	
%	16.20	2.60	13.50	3.10	3.40	24.50	4.90	19.40	2.90	15.60	3.20	
No	114	114	96	63	57	37	39	29	34	28	30	
Lymph node dissection												*P* < 0.001
Average No.	21.5	18.7	24.8	21.4	16.7	17.8	21	17.6	28.4	19.3	27.6	

### 2.3 患者术后短期疗效

术后短期疗效统计指标包括是否姑息性切除、切缘是否镜下无瘤残存、围手术期严重并发症发生率、术后30天内死亡率。各组间均存在统计学差异（表 3）。

**3:** 患者术后短期疗效 Post operation results of patients

Group	S1	S2	S3	S4	S5	S6	S7	S8	S9	S10	S11	Total
Number	136	117	111	65	59	49	41	36	35	32	31	714
R2 resection	9	5	11	4	3	4	1	2	1	1	0	*P* < 0.001
	6.60%	4.30%	9.90%	6.20%	5.10%	8.20%	2.40%	5.60%	2.90%	3.10%	0.00%	
R1 resection	0	0	1	2	1	2	2	0	0	1	0	*P* < 0.001
	0.00%	0.00%	0.90%	3.10%	1.70%	4.10%	4.90%	0.00%	0.00%	3.10%	0.00%	
Perioperative serious complications	4	2	5	2	3	4	1	2	1	3	0	*P* < 0.001
	2.90%	1.70%	4.50%	3.10%	5.10%	8.20%	2.40%	5.60%	2.90%	9.40%	0.00%	
Death within 30 Days	3	2	5	2	2	3	0	1	1	2	0	*P* < 0.001
	2.20%	1.70%	4.50%	3.10%	3.40%	6.10%	0.00%	2.80%	2.90%	6.30%	0.00%	

### 2.4 患者术后5年生存情况

如图 1A所示，不同医生手术患者之间生存情况存在明显差异（*P* < 0.01）。图 1B-图 1D展示不同临床分期患者生存情况，同样存在显著性差异。值得注意的是，在不同期别分析中，生存较好的组别具有一致性。

**1:**
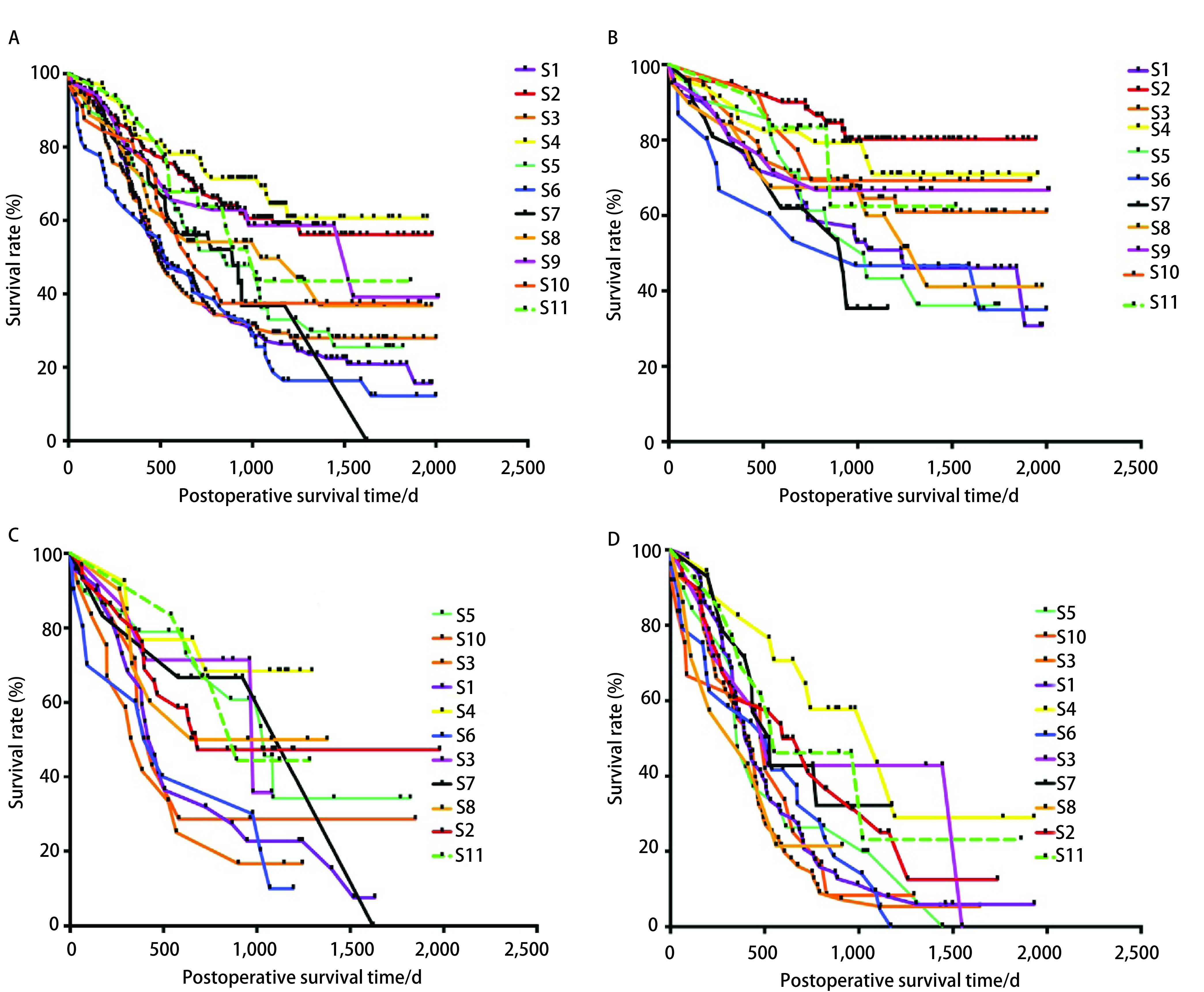
患者生存时间。A：全部患者；B：Ⅰ期患者；C：Ⅱ期患者；D：Ⅲ期患者。 The survival time of patients. A: All patients; B: Stage Ⅰ patients; C: Stage Ⅱ patients; D: Stage Ⅲ patients.

### 2.5 患者预后多因素相关性分析

在*Cox*回归多因素分析中，主刀医生是患者预后的独立影响因素（Sig=0.000），如表 4所示。影响预后的因素还包括：细胞分化、TNM分期、手术时间、是否根治性切除等（表 4）。

**4:** 患者预后多因素相关性分析（*Cox*回归） Analysis of 5-years survival

Variable	B	SE	Wald	df	Sig.	Exp(B)	95%CI for Exp(B)	
							Lower	Upper
Gender	-0.292	0.189	2.378	1	0.123	0.747	0.515	1.082
Smoke	0.118	0.172	0.47	1	0.493	1.125	0.803	1.576
Pathology	-0.188	0.128	2.18	1	0.14	0.828	0.645	1.064
Differentiation	-0.216	0.086	6.25	1	0.012	0.806	0.68	0.954
T	0.032	0.086	0.14	1	0.709	1.033	0.872	1.223
N	0.163	0.18	0.829	1	0.363	1.178	0.828	1.674
TNM	0.417	0.186	5.051	1	0.025	1.518	1.055	2.183
Surgeon	0.091	0.017	29.034	1	0	1.095	1.059	1.131
Operation type	0.051	0.054	0.905	1	0.342	1.052	0.947	1.169
Operation hours	0.242	0.11	4.822	1	0.028	1.273	1.026	1.58
R0 resection	0.66	0.172	14.672	1	0	1.935	1.38	2.712
TNM: tumor-node-metastasis.								

## 3 讨论

多项研究^[5-7]^表明在外科医生积累手术经验阶段，围手术期并发症发生率要显著高于有经验的医生，患者的术后生存也存在明显的差异。有研究^[8]^在分析了14.5万直结肠、食管、肺、乳腺、甲状腺等手术患者，发现有经验的医生进行手术的患者，术后并发症发生率和在院时间相比无经验的医生具有明显优势。Granero等^[9]^报道在医生的专业性会影响结肠癌患者的预后。Hillner等^[10]^也认为无论是医疗机构和医生的经验和专业都会影响肿瘤患者的预后。但是对于有经验的，专科医生之间的比较，尚未见到相关研究。

本研究纳入的患者都是经过有经验的肺癌专科医生进行的手术，所有手术方式都是遵循肺癌诊治规范指南进行，淋巴结清扫个数均达到16个以上。在这样基础条件下，我们可以观察到患者预后情况在组间存在着较大的差异，通过多因素回归分析，提示主刀医生是影响患者生存的独立因素。

这可能是由于术者技术水平和术中习惯造成的。典型的，各组间患者术中输血率最高24.5%，最低2.6%，相差近10倍。一方面可能是手术难度和技术差异造成术中失血量差别较大，另一方面，也可能是术中输血必要性和安全性观念差异较大造成的。诸如此类细节性差异不断累积，会形成对于患者预后的显著影响。

医生对于患者的选择性是客观存在的，部分医生倾向于治疗早期患者，可能会在全部患者生存中获得较好的表现。针对这个假设，我们对患者进行了分临床期别分析。组间差异在Ⅰ期、Ⅱ期、Ⅲ期中仍然存在。S4、S2、S11、S9组患者在各期别当中都获得了较好的生存；S1、S3、S5、S6组患者相比生存普遍较差。这种一致性说明了确实有特定均一的因素影响了各组别的患者预后。

另一值得注意的是，医生手术量对于患者预后的影响在本研究中并没有体现。患者数量较多的S1-S4组分别归类到预后较好的组别（S2、S4）和预后较差的组别（S1、S3）；数量最少的S11组获得了较好的生存。这说明，在技术成熟的，有经验的外科医生中，患者数量已经不成为影响技术水平的主要因素。

本研究存在一定的局限性。第一，本研究为回顾性研究，存在固有的缺陷，所获得的结论需要进行前瞻性大规模研究证实。第二，本研究纳入患者时间较为久远，不能代表现今手术治疗水平；但优势在于可以获得完整的五年生存时间以及在胸腔镜微创手术未能普及的年代，开胸手术术式具有相对的统一性。第三，本研究总体纳入患者数量712例，但由于术者较多，部分组别患者数差别较大；第四，本研究没有考虑术后辅助及复发后，综合治疗的影响，这一部分治疗肯定也会对患者生存造成影响。

本研究的临床意义在于揭示外科手术对于肺癌患者预后生存的巨大影响。更重要的是使外科医生认知到，医生间手术技术和水平的差别客观存在，并且将直接影响患者的长期生存。在精准医疗和大数据不断发展的情境下，以患者生存时间来判断外科医生水平的时代并不遥远，每一位外科医生都应该专注于治疗的长期结果，不断改进手术技术和理念。
